# Effect of Gamification With and Without Financial Incentives to Increase Physical Activity Among Veterans Classified as Having Obesity or Overweight: A Randomized Clinical Trial

**DOI:** 10.1001/jamanetworkopen.2021.16256

**Published:** 2021-07-09

**Authors:** Anish K. Agarwal, Kimberly J. Waddell, Dylan S. Small, Chalanda Evans, Tory O. Harrington, Rachel Djaraher, Ai Leen Oon, Mitesh S. Patel

**Affiliations:** 1 Department of Emergency Medicine, The Perelman School of Medicine, The University of Pennsylvania, Philadelphia; 2 The Penn Medicine Nudge Unit, The University of Pennsylvania, Philadelphia; 3 Crescenz Veterans Affairs Medical Center, Philadelphia, Pennsylvania; 4 Statistics Department,The Wharton School, The University of Pennsylvania, Philadelphia; 5 Health Care Management Department, The Wharton School, The University of Pennsylvania, Philadelphia

## Abstract

**Importance:**

Gamification is increasingly being used for health promotion but has not been well tested with financial incentives or among veterans.

**Objective:**

To test the effectiveness of gamification with social support, with and without a loss-framed financial incentive, to increase physical activity among veterans classified as having overweight and obesity.

**Design, Setting, and Participants:**

This 3-group randomized clinical trial had a 12-week intervention period and an 8-week follow-up period. Participants included veterans with a body mass index greater than or equal to 25 who were receiving care from a single site in Philadelphia, Pennsylvania. Participants underwent a remotely monitored intervention from March 19, 2019, to August 9, 2020. Data analyses were conducted between October 1, 2020, and November 14, 2020.

**Interventions:**

All participants received a wearable device to track step counts and selected a step goal. The control group received feedback from their devices only. Participants in the 2 gamification groups were entered into a 12-week game with points and levels designed using behavioral economic principles and selected a support partner to receive weekly updates. Participants in the loss-framed financial incentive group had $120 allocated to a virtual account and lost $10 if weekly goals were not achieved.

**Main Outcomes and Measures:**

The primary outcome was the change in mean daily steps from baseline during the intervention. Secondary outcomes include proportion of days goals were achieved and changes during follow-up.

**Results:**

A total of 180 participants were randomized, 60 to the gamification with social support group, 60 to the gamification with social support and loss-framed financial incentives group, and 60 to the control group. The participants had a mean (SD) age of 56.5 (12.9) years and a mean (SD) body mass index of 33.0 (5.6); 71 participants (39.4%) were women, 90 (50.0%) were White, and 67 (37.2%) were Black. During the intervention period, compared with control group participants, participants in the gamification with financial incentives group had a significant increase in mean daily steps from baseline (adjusted difference, 1224 steps; 95% CI, 451 to 1996 steps; *P* = .005), but participants in the gamification without financial incentives group did not (adjusted difference, 433 steps; 95% CI, −337 to 1203 steps; *P* = .81). The increase for the gamification with financial incentives group was not sustained during the follow-up period, and the step count was not significantly different than that of the control group (adjusted difference, 564 steps; 95% CI, −261 to 1389 steps; *P* = .37). Compared with the control group, participants in the intervention groups had a significantly higher adjusted proportion of days meeting their step goal during the main intervention and follow-up period (gamification with social support group, adjusted difference from control, 0.21 participant-day; 95% CI, 0.18-0.24 participant-day; *P* < .001; gamification with social support and loss-framed financial incentive group, adjusted difference from control, 0.34 participant-day; 95% CI, 0.31-0.37 participant-day; *P* < .001).

**Conclusions and Relevance:**

Among veterans classified as having overweight and obesity, gamification with social support combined with loss-framed financial incentives was associated with a modest increase in physical activity during the intervention period, but the increase was not sustained during follow-up. Gamification without incentives did not significantly change physical activity.

**Trial Registration:**

ClinicalTrials.gov Identifier: NCT03563027

## Key Points

**Question:**

What is the effect of gamification with social support and loss-framed financial incentives on the promotion of physical activity among veterans with overweight and obesity?

**Findings:**

In this randomized clinical trial of 180 veterans with overweight and obesity, gamification with social support and loss-framed financial incentives modestly increased physical activity during the 12-week intervention period, but the effect was not sustained during an 8-week follow-up period.

**Meaning:**

These findings suggest that gamification with social support, when combined with loss-framed financial incentives, can modestly increase physical activity among veterans with obesity and overweight, but future investigations should be conducted with a more representative sample of veterans and may need to be combined with other approaches to increase and sustain changes in physical activity.

## Teaser

This randomized clinical trial examines the effectiveness of gamification with social support, with and without a loss-framed financial incentive, to increase physical activity among veterans with overweight and obesity.

## Introduction

More than 80% of veterans have at least 2 risk factors for cardiovascular disease.^1^ Physical activity is associated with reduced risk for cardiovascular disease, yet less than one-half of veterans achieve enough activity to reduce their risk of the morbidity and mortality associated with cardiovascular disease.^2,3^ Motivating and sustaining regular physical activity is challenging for individuals.^4,5^ One promising approach has applied gamification, the use of game-design elements, to motivate behavior change.^6,7,8,9^ Workplace wellness programs have incorporated gamification with wearable devices to promote physical activity.^10,11,12^ The uptake of these methods is varied, and less is known about their effectiveness among veterans.^13^ Research^8,14^ has studied the effect of behavioral economic strategies on motiving healthy behaviors, yet these remained unexplored in veterans.

Two behavioral economics concepts can promote activity, including social incentives, or the influences that motivate using social ties and connections, and loss-framed financial incentives. Gamification with social incentives has been associated with significant increases in activity,^8,14^ and loss-framed financial incentives have been separately shown to be associated with increased physical activity.^15,16,17^ Gaps persist in understanding the effect of how these concepts could be combined in interventions specifically for veterans.

Our objective was to conduct a randomized clinical trial to test the effectiveness of gamification with social support, with and without loss-framed financial incentives, to promote physical activity among veterans classified as having overweight and obesity. The intervention period was 12 weeks, and participants were remotely monitored using wearable activity tracking devices.^18^

## Methods

### Study Design

This was a randomized clinical trial testing gamification with social support, with and without loss-framed financial incentives, vs usual care to promote physical activity among veterans classified as having overweight and obesity (body mass index [BMI], calculated as weight in kilograms divided by height in meters squared, ≥25). The trial period (March 19, 2019, to August 9, 2020) consisted of a 2-week baseline period, a 12-week intervention period, and an 8-week follow-up. The trial protocol (Supplement 1) was approved by the Corporal Michael J. Crescenz VA Medical Center’s institutional review board. This trial followed the Consolidated Standards of Reporting Trials (CONSORT) reporting guideline. All participants provided written informed consent and received $25 at enrollment, $50 upon completion, and a wearable device valued at $100.

The study used Way to Health (WTH),^18^ a platform at the University of Pennsylvania for remote monitoring and interventions.^8,14,15,16,19^ Participants used WTH to create an account, provide consent, and complete baseline assessments. Participants were mailed a wrist-worn wearable device (Alta or Inspire; both from FitBit) and selected a mode of study communication (email, text message, or both). Wearable device data were linked with WTH to allow for data sharing. Prior work^20,21^ has demonstrated consistent accuracy of wearable devices such as those used in this study for tracking step counts. Participants kept the device. Participants established a baseline step count and self-selected a step goal increase. Participants randomized to the gamification with social support and loss-framed financial incentive were eligible to receive an additional $120, which was placed into an account, and $10 per week was deducted if step goals were not met. At 20 weeks, participants completed a telephone experience survey.

### Participants

Eligible participants were aged 18 years or older, receiving care at the Corporal Michael J. Crescenz VA Medical Center, able to provide informed consent, interested in a 20-week study, had a BMI greater than or equal to 25, and owned a smartphone or tablet. Participants were excluded if there was a condition that made participation infeasible (eg, inability to provide informed consent), non-English speaking, if participation was unsafe (eg, pregnancy), or if they were enrolled in another study targeting activity. Individuals were identified from the health record. The study team mailed outreach to 10 046 individuals, and 677 were assessed for eligibility by telephone. Recruitment began on March 19, 2019, and the final participant completed the follow-up period on August 9, 2020. Participants were enrolled on a rolling basis when deemed eligible and after informed consent. Participants were not blinded to their study assignment group.

### Baseline Assessment and Goal Setting

Participants became accustomed to the device over 2 weeks and established a baseline step count using the second week of data.^8,14,15^ The first week was ignored to diminish the potential bias from higher activity during initial use. If less than 4 days’ worth of data were available during the second week (13 participants), the participant was contacted and the period was extended until at least 4 days’ worth of data were captured. Similar to prior research,^8,14,22^ participants began with goal setting for step count increase (33%, 40%, 50%, or any goal ≥1500 steps above baseline). Participants were told to strive for goals during the intervention period and follow-up.

### Randomization

After baseline establishment, participants were randomized electronically using block sizes of 3 across groups. All investigators and data analysts were blinded until the study and analysis were completed.

### Interventions

Control participants received feedback and goal-setting from the device only. Upon completion of the 2-week baseline period, participants in the intervention groups were entered into an automated game with points and levels (participants did not have to actively play the game, just strive for their goals) and were provided a daily progress notification. The design was based on prior work^8,14,22^ incorporating behavioral economic principles. First, participants signed a precommitment pledge to strive for goals.^23,24^ In prior work,^8^ immediately activated goals were difficult for participants, and, thus, this trial included a 4-week ramp-up period in which step goal targets increased by 25% each week to the final goal.^14^ Participants were asked to strive for their goal but could change the goal if desired within the options provided.

Second, participants received 70 points per week (10 per day). If the participant did not achieve their goal, they lost 10 points. This leverages loss aversion, which has been demonstrated to motivate behavior more effectively with losses rather than gains.

Third, participants could move up or down levels on the basis of weekly point totals (from lowest to highest: blue, bronze, silver, gold, and platinum). This creates goal gradients, a sense of status, and progression. If participants had at least 40 weekly points, they would move up. We leveraged the fresh start effect, starting each week with a fresh set of 70 points; this effect describes the tendency for aspirational behavior around temporal landmarks such as the beginning of the year, month, or week.^25^

Fourth, participants in the interventions identified a social support sponsor. This sponsor was encouraged to support the participant, but no specific training was given to the sponsor. A weekly report was emailed to the sponsor with the participant’s performance—step goal, mean step count for that week, points, and level.

In the loss-framed financial incentive group, participants played the game and had $120 placed in a virtual account. Each week if the participant did not reach their goal, $10 was deducted. This leverages prior work^15,16^ demonstrating that loss-framed financial incentives can be used to increase physical activity.

### Outcome Measures

The primary outcome was change in mean daily steps from baseline during weeks 5 to 12 of the intervention period (excluding the 4-week ramp-up period). Secondary outcomes measures included change in mean daily steps from baseline during the follow-up period (weeks 13-20) and the proportion of participant-days that step count was achieved during the intervention and follow-up periods.

### Statistical Analysis

This study was powered a priori for 2 phases of hypothesis testing. In the first phase, the change in daily steps for each intervention was compared with control. We estimated that a sample of 180 participants (60 per group) would ensure 80% power to detect a 900-step difference, a value informed by prior research,^8,14^ between the control and each of the intervention groups, with an SD of 1500 steps and a 1:1:1 allocation ratio. This calculation assumed a 10% missing data rate and a conservative Bonferroni adjustment of the type I error rate with a 2-sided α = .025. In the second phase, we compared each intervention group with each other only if they were both significantly different than control, with a conservative Bonferroni adjustment of the type I error rate with a 2-sided α = .025 to adjust for up to 3 comparisons.

After randomization, 2 participants were deemed ineligible and were not started in the intervention because of a technical issue with WTH. All other randomly assigned participants were included in the modified intention-to-treat analysis. Daily steps were obtained as continuous variables (participant-day level). The daily step data were dichotomized into a binary variable that indicated whether a participant met their goal for each day of the study. This binary variable was used to examine the proportion of days participants achieved their goal.

Data were missing for any day the participant did not upload their step data or wear their device or had a daily step count that was less than 1000 steps, because previous research^26,27^ indicated that values less than 1000 may represent incomplete data capture. Missing data rates during the intervention period were between 14.6% and 19.3% (eTable 1 in Supplement 2), which were consistent with previous studies.^14,15^ We used multiple imputation for step values that were missing or less than 1000 steps per day.^8,14,15^ Five imputations were completed using the mice package in R statistical software version 4.0.2 (R Project for Statistical Computing),^28,29^ and the following covariates were included: participant random effect, study week, calendar month, baseline steps, age, sex, self-identified race/ethnicity, educational level, marital status, annual household income, study group, and BMI. Race/ethnicity was evaluated in this study because the veteran population is diverse. All model results were pooled using Rubin standard rules.^30^ Sensitivity analyses were conducted using the collected step data both with and without steps less than 1000.

Unadjusted mean daily steps were calculated by week for each group. For the adjusted analysis, changes in daily steps from baseline during the intervention (weeks 5-12) and follow-up (weeks 13-20) periods were examined using a generalized linear mixed effects model adjusting for participant random effect and calendar month, baseline steps, and study group as fixed effects. Both intervention groups were compared with control. We obtained differences in daily steps using the least squared means command for each intervention group. Adjusted differences in the proportion of days participants met their step goal in each intervention group, compared with control, were evaluated using the bootstrap method. Participants within each group were resampled 500 times. The model was adjusted for a participant random effect, with calendar month and study group as fixed effects. All analyses were completed in R statistical software version 4.0.2 (R Project for Statistical Computing) using the lme4 package.^31^ Data analyses were conducted between October 1, 2020, and November 14, 2020.

## Results

**Figure 1.**
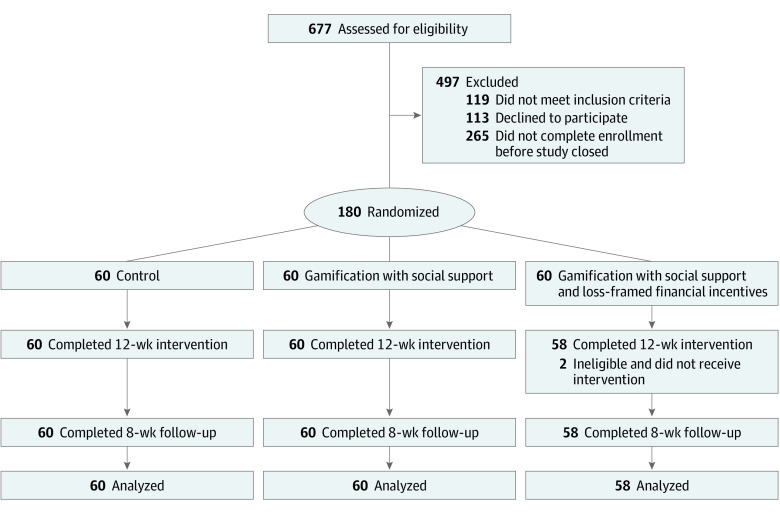
CONSORT Diagram Participants in all groups received a wearable device and established baseline measures. Participants in the control group received regular feedback from the wearable device, but no other interventions. Participants in the gamification groups set goals for daily step counts and were entered into 1 of 2 gamification interventions that ran automatically for 20 weeks. Because of a technical issue with the platform, 2 participants in the gamification group with social support and loss-framed financial incentives were randomized but were not eligible and therefore did not receive the intervention.

Of 10 046 veterans invited to participate, 677 (6.7%) were assessed for eligibility, and 180 (1.8%) were randomized, 60 to the gamification with social support group, 60 to the gamification with social support and loss-framed financial incentives group, and 60 to the control group (Figure 1). Participants had a mean (SD) age of 56.5 (12.9) years, mean (SD) weight of 217.3 (41.7) pounds (98.6 [18.9] kg), and mean (SD) BMI of 33.0 (5.6); 71 participants (39.4%) were women, 90 (50.0%) were White, and 67 (37.2%) were Black. Participant characteristics were well balanced across the study groups with no significant differences in age, weight, BMI, sex, or race/ethnicity (Table 1). Mean (SD) baseline daily steps were 5881 (2038) in the control group, 6012 (2494) in the gamification with social support group, and 6105 (2320) in the gamification with social support and loss-framed financial incentive group.

**Table 1.** Characteristics of Study Participants

Characteristic	Participants, No. (%)		
	Control (n = 60)	Gamification	
		Social support (n = 60)	Social support and loss-framed financial incentive (n = 60)
Sociodemographic characteristics			
Age, mean (SD), y	58.1 (12.6)	53.7 (13.0)	57.9 (12.8)
18-35	2 (3.3)	6 (10.0)	4 (6.7)
36-50	16 (26.7)	21 (35.0)	13 (21.7)
51-64	21 (35.0)	17 (28.3)	22 (36.6)
≥65	21 (35.0)	16 (26.7)	21 (35.0)
Sex			
Female	22 (36.7)	29 (48.3)	20 (33.3)
Male	32 (53.3)	31 (51.7)	40 (66.7)
Race/ethnicity			
Non-Hispanic			
White	36 (60.0)	25 (41.7)	29 (48.3)
Black	18 (30.0)	26 (43.3)	23 (38.4)
Asian	1 (1.7)	1 (1.7)	0
Hispanic	5 (8.3)	3 (5.0)	5 (8.3)
Other^a^	0	5 (8.3)	3 (5.0)
Education			
High school graduate or general equivalency diploma	10 (16.7)	4 (6.7)	11 (18.3)
Some college or specialized training	25 (41.7)	23 (38.3)	17 (28.3)
College graduate	25 (41.7)	33 (55.0)	32 (53.3)
Martial status			
Single	12 (20.0)	17 (28.3)	17 (28.3)
Married	36 (60.0)	26 (43.3)	26 (43.4)
Other	12 (20.0)	17 (28.3)	17 (28.3)
Annual household income, $			
<50 000	23 (38.3)	25 (41.7)	18 (30.0)
50 000-100 000	26 (43.3)	22 (36.6)	31 (51.7)
>100 000	11 (18.3)	12 (20.0)	11 (18.3)
Missing	0	1 (1.7)	0
Employment status			
Full-time	18 (30.0)	22 (36.7)	25 (41.7)
Part-time	4 (6.7)	2 (3.3)	4 (6.7)
Not employed	38 (63.3)	36 (60.0)	31 (51.7)
Military service period^b^			
Between the Korean conflict and the Vietnam era	1 (1.7)	0	1 (1.7)
Vietnam (1961-1975)	19 (31.7)	15 (25.0)	24 (40.0)
Post-Vietnam	27 (45.0)	25 (41.7)	22 (36.7)
The Gulf War (1990-1991)	19 (31.7)	15 (25)	15 (25.0)
1991-2001	28 (46.7)	25 (41.7)	27 (45.0)
2001-2011	23 (38.3)	19 (31.7)	18 (30.0)
After 2011	14 (23.3)	10 (16.7)	7 (11.7)
Baseline outcome measures, mean (SD)			
Baseline weight, lb	216.7 (41.9)	218.1 (47.8)	217.1 (35.2)
Body mass index^c^	32.6 (5.1)	33.7 (6.3)	32.8 (5.4)
Baseline step count	5881 (2038)	6012 (2494)	6105 (2320)
Step goal selection, increase from baseline, %			
33	25 (41.7)	28 (46.7)	20 (33.3)
40	9 (15.0)	9 (15.0)	11 (18.3)
50	5 (8.3)	7 (11.7)	10 (16.7)
Other self-selected	21 (35.0)	16 (26.6)	19 (31.7)

SI conversion factor: To convert pounds to kilograms, multiply by 0.45.

^a^ Other indicates Native American, Alaska Native, or more than 1 race.

^b^ Military service periods are not mutually exclusive.

^c^ Body mass index is calculated as weight in kilograms divided by height in meters squared.

### Daily Step Counts

**Figure 2.**
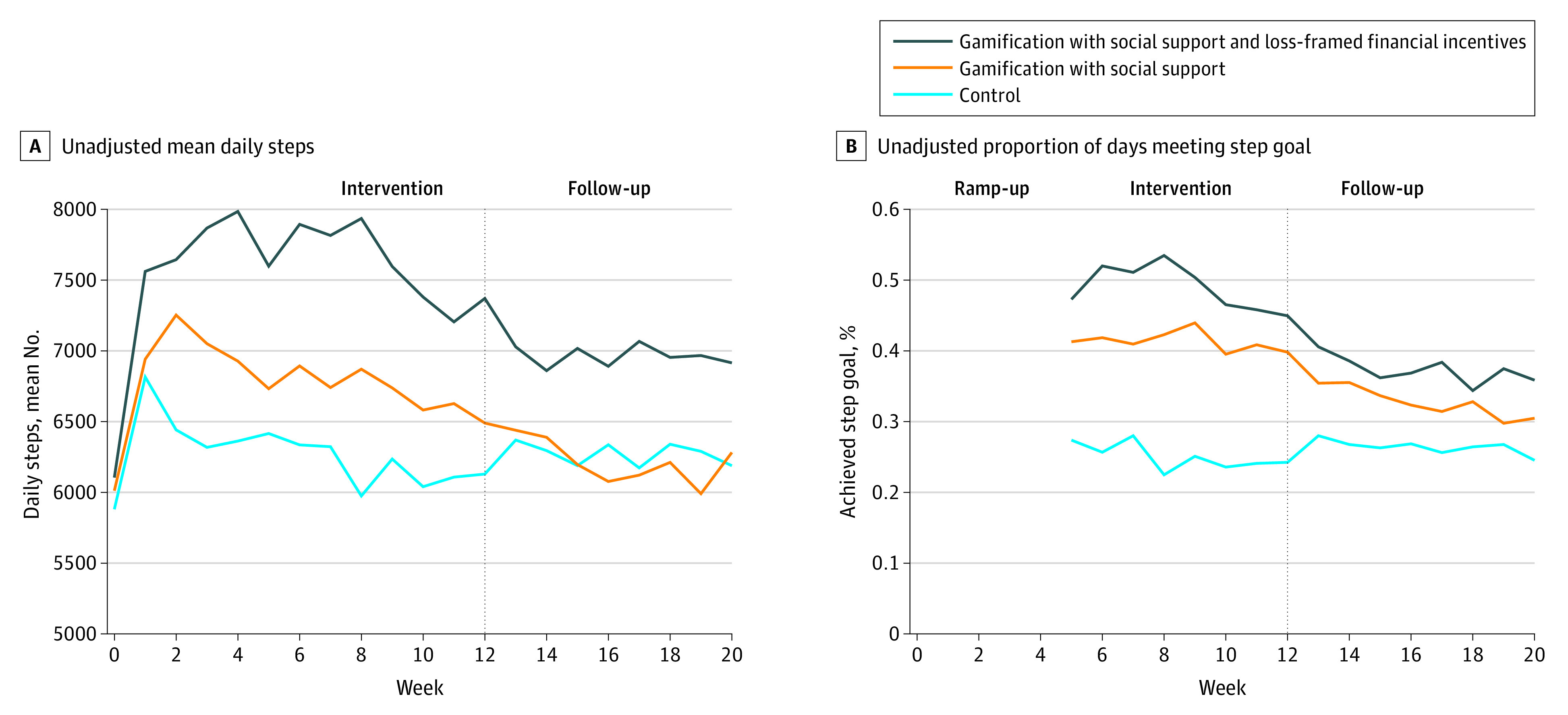
Unadjusted Outcomes Depicted are outcome measures using imputed data as the unadjusted mean daily steps for each group by week (A) and mean proportion of participant-days meeting the step goal (B). The 4-week initial ramp-up period where daily step goal targets increased by 25% per week from baseline to full goal represents time when individuals in the intervention groups could not achieve full step goal until week 5.

The unadjusted mean daily steps by week and group are depicted in Figure 2A. The mean (SD) baseline step count in the control group was 5881 (2038) steps and increased to 6484 (3704) steps during the ramp-up. This declined to near baseline by week 8 (5974 [3735] steps). The mean (SD) baseline step count in the gamification with social support group increased from 6012 (2494) steps to 7043 (3841) steps (difference, 1031 steps) during the ramp-up, remained at 6870 (3889) steps until week 8, and declined during follow-up (6213 [3666] steps). Mean (SD) daily steps in the gamification with social support and loss-framed financial incentives group increased from a baseline of 6105 (2320) steps to 7764 (3716) steps (difference, 1659 steps) and remained steady until week 8. The mean step count decreased over the main intervention period and remained at 6962 (3888) steps during the follow-up period.

In the adjusted model, participants had a significantly greater increase (approximately 20%) in mean daily steps during the main intervention in the gamification with social support and loss-framed financial incentive group compared with the same period in the control group (adjusted difference, 1224 steps; 95% CI, 451 to 1996 steps; *P* = .005). There was no significant difference in the gamification with social support group (433 steps; 95% CI, −337 to 1203 steps; *P* = .81). There was no sustained significant increase during the follow-up period in the gamification with social support group (−160 steps; 95% CI, −983 to 663 steps; *P* = .92) or the gamification with social support and loss-framed financial incentive group (564 steps; 95% CI, −261 to 1389 steps; *P* = .37) (Table 2). Results were similar in sensitivity analyses that used collected data without multiple imputation (eTable 2 in Supplement 2).

**Table 2.** Adjusted Differences in Daily Steps Among the Study Groups

Variable	Control	Gamification	
		Social support	Social support and loss-framed financial incentives
Steps per day at baseline, mean (SD), No.	5881 (2038)	6012 (2494)	6105 (2320)
Main intervention period^a^			
Steps per day, mean (SD), No.	6195 (3767)	6709 (3827)	7599 (3897)
Main adjusted model^b^			
Difference vs control and adjusted for baseline (95% CI)	NA	433 (−337 to 1203)	1224 (451 to 1996)
* P* value	NA	.81	.005
Follow-up period^a^			
Steps per day, mean (SD), No.	6272 (3839)	6213 (3666)	6962 (3888)
Main adjusted model^b^			
Difference vs control and adjusted for baseline (95% CI)	NA	−160 (−983 to 663)	564 (−261 to 1389)
* P* value	NA	.92	.37

Abbreviation: NA, not applicable.

^a^ The main intervention period included weeks 5 to 12 and excluded the ramp-up phase. The follow-up period included weeks 13 to 20.

^b^ The main adjusted models use imputed data and adjust for baseline outcome measure and calendar month.

### Proportion of Participant-Days Goal Achieved

The unadjusted proportion of participant-days when step goals were achieved in the main intervention period were 0.25 in the control group, 0.41 in the gamification with social support group, and 0.48 in the gamification with social support and loss-framed financial incentive group (Table 3). These levels were lower during the follow-up period, at 0.26 participant-day in the control group, 0.32 participant-day in the gamification with social support group, and 0.37 participant-day in the gamification with social support and loss-framed financial incentive group.

**Table 3.** Adjusted Differences in the Proportion of Participant-Days That Step Goals Were Achieved

Variable	Control	Gamification	
		Social support	Social support and loss-framed financial incentives
Main intervention period^a^			
Proportion of participant-days that step goal was achieved	0.25	0.41	0.48
Main adjusted model^b^			
Difference vs control (95% CI)	NA	0.21 (0.18-0.24)	0.34 (0.31-0.37)
*P* value	NA	<.001	<.001
Follow-up period^a^			
Proportion of participant-days that step goal was achieved	0.26	0.32	0.37
Main adjusted model^b^			
Difference vs control (95% CI)	NA	0.09 (0.06-0.10)	0.18 (0.15-0.20)
*P* value	NA	<.001	<.001

Abbreviation: NA, not applicable.

^a^ The main intervention period included weeks 5 to 12 and excluded the ramp-up phase. The follow-up period included weeks 13 to 20.

^b^ The main adjusted models use imputed data and adjust for baseline outcome measure and calendar month.

In adjusted models, compared with controls, participants had a significantly higher proportion of days meeting their step goal during the intervention period (Figure 2B) in the gamification with social support group (adjusted difference from control, 0.21 participant-day; 95% CI, 0.18-0.24 participant-day; *P* < .001) and in the gamification with social support and loss-framed financial incentive group (0.34 participant-day; 95% CI, 0.31-0.37 participant-day; *P* < .001). The proportion of days meeting the step goal were modestly higher in the follow-up period in the gamification with social support group (0.09 participant-day; 95% CI, 0.06-0.10 participant-day; *P* < .001) and in the gamification with social support and loss-framed financial incentive group (0.18 participant-day; 95% CI, 0.15-0.20 participant-day; *P* < .001).

### Safety and Patient Experience

At the end of the trial, 91% of participants (164 of 180 of participants) completed the survey. A majority (129 of 164 participants [79%]) reported satisfaction with the wearable device. The overall rate of satisfaction with participating in the study was 81% (133 of 164 participants), and 68% (112 of 164 participants) reported that the study helped to increase physical activity (eTable 3 in Supplement 2). There were no adverse events.

## Discussion

In this randomized clinical trial of veterans classified as having overweight and obesity, we found that gamification with social support was associated with modestly increased physical activity compared with control during the intervention period when combined with loss-framed financial incentives. The magnitude of effect was approximately 1200 steps per day (1031 steps per day in the gamification with social support group and 1659 steps per day in the gamification with social support and financial incentives group), or 50 miles per individual over the intervention period (estimated as 2000 steps per mile). Recent evidence and guidelines^32,33^ indicate that small increases in activity provide health benefits. During the follow-up, the change in step counts compared with control decreased to 564 steps and was not significantly different. Gamification without incentives was not significantly different in either period. To our knowledge, this is one of the first trials to test these behavioral economic approaches among veterans.

These findings expand on the literature of using gamification, social support incentives, and loss-framed financial incentives to motivate activity and reveal insights for the design of future interventions for veterans.^8,14,15^ First, this trial tested strategies from behavioral economics (eg, precommitment, gamification, loss aversion, goal gradients, and the fresh start effect) for increasing physical activity and monitoring it remotely. Gamification has been used in different modalities and has demonstrated varying effectiveness.^10,11,21,34^ Less is known about its effectiveness on activity for veterans. Our findings suggest that these approaches can motivate physical activity over shorter-term periods, but additional work needs to focus on increasing the effect and sustaining it. A growing body of research is testing various social supports to help motivate activity, and this trial demonstrates findings consistent with those of other trials.^14^

Second, participants in intervention groups had an increase in mean daily step counts during the intervention period but not during the follow-up period. The gamification with social support had an unadjusted mean daily increase of 433 steps, and the gamification with social support and loss-framed financial incentives had an increase of 1224 adjusted steps per day. The results for the gamification with social support group were not significant, but they reflect results consistent with prior work.^14,15^ It is important to highlight that the social support used in this trial—a weekly email—was passive. Another recent study^35^ that tested gamification with social support after discharge from the hospital did not lead to changes in physical activity. It may be that these types of approaches are not impactful in the test patient populations. Future studies testing more intensive social incentives, such as team-based cooperation or competition, may demonstrate larger or longer effects.^22^

Third, loss-framed financial incentives may play a role in motivating activity. During the intervention period, participants in the gamification with social support and loss-framed financial incentive group had a mean adjusted increase in daily step counts by approximately 20%. This represents a higher value compared with prior trials (10%-15%).^8,14^ To our knowledge, there are no other veterans’ data with which to compare ours, and research indicates that even modest activity contributes to health.^32,33,36,37^ Components of financial incentive design may affect behavior and should be studied. Varying the interval to achieve goals (daily or weekly), the duration of the intervention, or the magnitude of the deduction may have different effects. The loss-framed incentive did not specifically incorporate other theories such as the fresh start effect (resetting the monetary amount)^25^ or immediate gratification.

### Strengths and Limitations

This study has strengths. The intervention is among the first to test behaviorally designed gamification, loss-framed financial incentives, and remote monitoring to track activity. The sample was unique because there were low baseline activity levels and it was diverse in terms of race/ethnicity, education, and income. The program used an automated platform and was remotely monitored, which makes it scalable. The study builds on literature studying mechanisms for promoting activity, and although the effects were not sustained, the findings highlight the effect of combining gamification with financial incentives to increase activity by 20%. Future studies should explore how to sustain these changes, how to increase their magnitude, and the varying effect across patients.

This study also has limitations. First, participants were from a single VA system and needed access to a smartphone or tablet, which may limit generalizability. However, more than 80% of US adults now own a smartphone.^38^ Second, among the 10 046 veterans invited by mail, only 6.7% expressed interest and only 1.8% were ultimately enrolled. These participants had higher than average step counts at baseline and may not be representative of the ideal target population for this type of intervention. Third, we evaluated activity using step counts and did not have data on other measures of activity. Fourth, the study found a significant increase in a single intervention group only, and given the study protocol, we were unable to compare across intervention groups. Fifth, longer-term evaluations are needed including investigations on metrics such as weight loss. Sixth, the effect of increased step counts remains understudied and future efforts will need to investigate these directly. There may be bias introduced by the support partner because there was no structured training for these individuals. Seventh, the population was largely male with a baseline step count near 6000, which may not be generalizable.

## Conclusions

In a randomized clinical trial of veterans classified as having overweight and obesity, gamification with social support when combined with loss-framed financial incentives modestly increased physical activity during the intervention, but changes were not sustained during follow-up. Gamification without financial incentives did not significantly change activity. Future interventions should be tested among a more representative sample of veterans and may need to be combined with other approaches to lead to larger and sustained changes in physical activity.

## Notes

Supplement 1. Trial Protocol and Statistical Analysis Plan

Supplement 2. **eTable 1.** Missing Data Rates by Arm and Study Period **eTable 2.** Adjusted Differences in Outcomes Without Multiple Imputation **eTable 3.** Patient Perceptions of Trial Experience From End-of-Study Surveys

Supplement 3. Data Sharing Statement
