# Supplementary material for: Effect of Gamification With and Without Financial Incentives to Increase Physical Activity Among Veterans Classified as Having Obesity or Overweight: A Randomized Clinical Trial
Source: JAMA Netw Open. 2021 Jul 9;4(7):e2116256. doi: 10.1001/jamanetworkopen.2021.16256 (PMC8271358; doi:10.1001/jamanetworkopen.2021.16256)
Supplement: Supplement 1. — Trial Protocol and Statistical Analysis Plan [file jamanetwopen-e2116256-s001.pdf]

SUPPLEMENT 1

Social Incentive-Based Gamification with and without Financial Incentives to Increase Physical Activity Among Overweight and Obese Veterans

Study Protocol

September 1, 2017

Section 1. General Information

Protocol Title: A Randomized Trial of Social and Financial Incentives to Increase Physical Activity Among Overweight and Obese Veterans

CMCVAMC Protocol Version Number and Date: Version 5, October 25, 2019

Principal Investigator (PI) Name: Mitesh Patel, MD, MBA, MS

PI's Academic Degree(s): *MD*

Is the study funded? *YES* **If "yes", specify funding agency:** *VA HSR&D, VA VISN CPPF Grant*

Is a grant application requesting funds for the study currently being reviewed? *NO*

CMCVAMC is the only institution involved: *YES*

CMCVAMC is the coordinating center in which the PI is the lead investigator: *NO*
If this answer is yes, complete the next two sections:

- **List the name(s) of the other site(s) involved**.
- **Provide the FederalWide Assurance (FWA) numbers for each site**.

State name of coordinating center if this is not CMCVAMC.

Describe PI's qualifications to conduct this project, and attach a copy of PI's VA or NIH biosketch. Be specific in regard to PI's research experience. Mitesh Patel, MD, MBA, MS is the Principal Investigator (PI) and is Core Investigator at the VA Center for Health Equity Research and Promotion (CHERP) and a staff physician at the CMCVAMC in Philadelphia. He is also an Assistant Professor of Medicine and Health Care Management at the Perelman School of Medicine and the Wharton School at the University of Pennsylvania. He has led more than 15 randomized clinical trials including many physical activity behavioral interventions that use the Way to Health research information technology platform. He has experience and training in behavioral economics, clinical trial design and analysis, health services research, and statistical analysis. He currently spends 80% of his effort on research and 20% on clinical and teaching activities.

Does any research staff member have an actual and/or perceived conflict of interest with this study? *NO*
If yes, explain.

Is this study a clinical trial? *YES* **If yes, specify the type.** *Phase III*

State the estimated length of time to complete enrollment of subjects. *6-9 months*

State the expected duration of participation by individual subjects (including any follow-up, e.g., need to re-contact subject for follow-up questions prior to closure of the study). *20 weeks*

Specify the projected date of completion of the study. *12/31/2019*

Section 2: Participating Site Specifications

2.1. **Where will the research project be conducted? (Check all that apply)**

☐ **VA Inpatient Setting** ☐ **VA Outpatient Clinic/Office**

☐ **VA Laboratories** **x Subject Homes**

☐ **University of Pennsylvania** ☐ **Community Based Outpatient Clinics (CBOCs)**

☐ **Other *(Specify)*:** [ ]

2.2. **If research is conducted at a non-VA site, please specify where and how much of the project will be conducted at that location**. This project will remotely track veterans' physical activity with the use of a wearable device, either a Fitbit Alta or Fitbit Inspire.

<u>Section 3: Introduction</u>

3.1. **Provide scientific background and rationale for study. Including summary of gaps in current knowledge, relevant data, and how the study will add to existing knowledge.** Over 80% of veterans have at least two risk factors for cardiovascular disease (CVD). Regular physical activity is associated with reduced risk for CVD, but less than half of veterans achieve enough physical activity to obtain these benefits. Digital health approaches that use engagement strategies such as gamification are commonly found within workplace wellness programs and mobile applications, but the evidence is limited. In 2015, the Veterans Affairs Evidence-Based Program published a systematic review which found only 14 trials on the use of wearable device technology with none that enrolled veterans for the purpose of testing how these devices could augment physical activity. Our prior work has demonstrated that wearables may be appropriate for monitoring health behaviors, but they alone they do not drive behavior change.

Social incentives or those influences that impact individuals to adjust their behaviors based on social ties and connections have been demonstrated in retrospective studies to influence behavior but have not been well examined prospectively. Insights from behavioral economics can be used to design gamification interventions to enhance social incentives such as the support, competition, or collaboration but the optimal design to increase physical activity is unknown. This study will use behavioral economics frameworks to design and test a social incentive-based gamification intervention with and without financial incentives to increase physical activity among overweight and obese veterans. We will test whether this intervention can be conducted remotely, thereby reducing participant burden of coming in for an in-person visit and creating a more scalable intervention design. Specifically, we will test the ability of using wearable devices with social and financial incentive-based interventions to augment physical activity as measured by change in mean daily step counts which will be tracked by a wearable activity tracker.

<u>Section 4: Objectives Section</u>

4.1. **Describe the study's purpose, specific aims, or objectives.**

The objective of this study is to use a randomized, controlled trial to test the effectiveness using a remotely-monitored social incentive-based gamification intervention with and without financial incentives to increase physical activity among overweight and obese veterans. All participants will use wearable devices to establish a baseline step count and monitor their physical activity during the study.

Primary outcome: The primary outcome is change in mean daily steps from baseline to the weeks 5 to 12 of the intervention period (which excludes the 4-week ramp-up phase).

Secondary outcomes: Secondary outcomes include change in mean daily steps from baseline to the 8-week follow-up period, Proportion of participant-days achieving step goals during the intervention and follow-up periods.

Exploratory outcomes: We will explore how participant characteristics and behaviors are associated with strong or poor physical activity performance.

4.2. **State the hypotheses to be tested.**

Hypothesis: The combination of social and financial incentives will lead to greatest increase in mean step counts from baseline.

Section 5: Study Procedures

5.1. Study Design

5.1.1. Describe in detail the experimental design, i.e. from recruitment procedures to study closure.

Design: This is a three-arm randomized, controlled trial with a 12-week intervention period and 8-week follow-up period. The study will be conducted using Way to Health (WTH), a research information technology platform at the University of Pennsylvania that has been used previously at the CMCVAMC for a behavioral study.

Study duration: This study is anticipated to take up to 2 years to complete and includes a 12-week intervention period and 8-week follow-up period.

Target population: Veterans, age 18 years or older, who received care at the CMCVAMC and have a body mass index of 25 or greater.

Accrual: This study has been powered for two phases of hypothesis testing. In the first phase, we will compare each of the two intervention arms to control. We estimate that a sample of 180 participants allocated in a 1:1:1 distribution, will ensure at least 80% power to detect a 900-step difference between each intervention arm and control, with a standard deviation of 1500 steps. This calculation assumes a 10% missing data rate and a conservative Bonferroni adjustment of the type I error rate with a 2-sided alpha of 0.025. In the second phase, we will compare successful intervention arms to each other. We expect that the magnitude of difference between intervention arms will be less than that of successful intervention arms compared to control. For this second phase of analyses will use a conservative Bonferroni adjustment of the type I error rate with a 2-sided alpha of 0.017 to adjust for up to 3 comparisons. In 2012, more than 57,500 veterans enrolled for care at CMCVAMC and comprised nearly 463,000 visits. Since 70% of veterans are overweight or obese, nearly 40,000 veterans may be eligible for this study. Based on this data and prior studies, we estimate that we can fill the study within 6 months. We will plan to oversample women and minorities in the recruitment process.

Interventions:
In order to minimize cost, time and travel for Veterans and to make study the least burdensome as possible, recruitment and enrollment will be completed remotely. Veterans will be asked to verify their identity using their name and age. Participants will be enrolled remotely using a standardized eligibility survey which will be conducted over the phone. Once deemed eligible and oral consent is obtained, participants will be asked to complete a telephone survey to obtain demographic data, activity data and information on use of technology. Participants will be told to wear the activity tracking device during day and night to get accustomed to it during a 2-week run-in period. Data collected from this time will be used to estimate a baseline step count using methods from prior work by using data from the second week (days 8 to 14), ignoring values less than 1000 steps (since evidence suggests these values are unlikely to represent actual activity). Participants without at least 4 days of data will be called to inquire if there are any issues with using the device and the period will be extended until at least 4 days of data are available to estimate a baseline step count.

Participants that have been confirmed by the study team to have an appropriate baseline step count will be called and asked to select a step goal increase as follows:

Goal Setting: Each participant will be asked to choose a step goal increase that is between 33% and 50% higher than their baseline (each step goal will be rounded up to the nearest hundred). A participant may also select to choose another goal as long as it is at least 1500 steps greater than his or her baseline.

Randomization: Participants are considered ready to be randomized once they have completed all surveys, established a baseline step count, and selected a step goal increase. Participants will then be randomly assigned in blocks of three. Participants in all arms will be asked to complete end of study surveys at 20 weeks on their experience in the study. The interventions within each arm are as follows:

Arm 1: Control

Participants in this arm will receive no other interventions during the 20-study.

Arm 2: Social incentive-based gamification intervention

Participants randomized to Arm 2 will play a game designed to leverage insights from behavioral economics and to enhance *supportive* social incentives.

At the beginning of each week the participant receives 70 points (10 for each day that week). If the participant does not achieve their step goal, they lose 10 points from their balance. This leverages loss aversion, which has been demonstrated to motivate behavior change more effectively with losses than gains. At the end of each week if the participant has at least 40 points, he or she will move up a level (levels from lowest to highest: blue, bronze, silver, gold, platinum). If not, participants will drop a level. All participants begin at the silver level. Each week, participants get a fresh set of 70 points on Monday.

Each participant will identify a family member or friend as a support sponsor. This sponsor will be encouraged to support the participant in their progress during the study. A weekly report will be sent by email to the sponsor with the participant's performance (e.g., step goal, average step count for that week, points and level).

Arm 3: Supportive social incentive intervention plus financial incentive

Participants randomized to Arm 3 will play the same game as in Arm 2 but will also have $120 placed in a virtual account. Each week if the participant reaches a higher level, having at least 40 points, they will keep the money in the account. If not, then $10 will be deducted. This will leverage prior work demonstrating that loss framed financial incentives can be used to increase physical activity. A participant in Arm 3 must complete the full course of the study intervention timeline to obtain any of this amount (up to $120).

5.1.2. **What research methods will be used in the project*? Check all that apply.***

**X Surveys/Questionnaires**	☐ **Interviews**	☐ **Audio Taping**
☐ **Behavioral Observations**	☐ **Chart Reviews**	☐ **Video Taping**
☐ **Focus Groups**	**X Randomization**	☐ **Double-Blind**
**X Control Group**	☐ **Placebo**	☐ **Withhold/Delay Treatment**
☐ **Specimen Collection**	☐ **Deception**	**X Telephone Survey**

☐ **Other (Describe)** Physical activity tracking via a wearable device.

5.1.3. **Provide description of the study population (delineate all categories of subjects – male, female, inpatients, outpatients, providers, family members, employees, etc.). Include anticipated initial enrollment numbers (and number of subjects anticipated to complete all aspects of the protocol).**

The study population will include 180 participants total: 1) adults age 18 years or older; 2) interest in participating in a 20-week study using wearable devices to track step counts and increase physical activity; 3) body mass index of 25 or greater; 4) Smartphone or tablet compatible with application for the wearable activity tracking device.

5.1.4. **As applicable, provide rationale and information on any added protections and safeguards for vulnerable populations (children, prisoners, pregnant women, physically or mentally-disabled persons, and economically or educationally disadvantaged persons).**

This will not include children or prisoners. For other vulnerable populations, this study will only include subjects wishing to participate by wearing a physical activity tracking device. Those

individuals with physical disability which prevents them from wearing the device or for its proper tracking will not be included in the study.

5.1.5. Does this project target a specific race or ethnic group as subjects? *NOT APPLICABLE*
If yes, check all that apply.

Race

☐ **American Indian or Alaska Native**
☐ **Asian**
☐ **Black or African American**
☐ **Native Hawaiian or other Pacific Islander**
☐ **White**
☐ **Other** [ ]

Ethnicity

☐ **Hispanic or Latino**
☐ **Not Hispanic or Latino**

5.1.6. Will this study bank/store specimens for future research? *NO*

5.1.6.1. **If yes, include information on specimens to be banked/stored.**

5.1.6.2. **If specimens will be banked/stored, specify location.**

5.1.6.3. **If the location of the specimen bank is a non-VA site, has the mandatory approval from VA Central Office been obtained through submission of a tissue banking application?** *Choose an item.*

5.1.6.3.1. **If yes, provide a copy of the response from VA Central Office.**

5.1.6.3.2. ***IF BANKING SPECIMENS, IT MUST BE AT A VA APPROVED FACILITY. (For additional information, go to the following website http://www.research.va.gov/programs/tissue_banking/, or contact the IRB office.)***

5.1.6.4. **If applicable, explain how destruction of banked samples will be substantiated.**

5.1.6.5. **Do you anticipate using the banked specimens for other studies beyond the defined study period and defined study parameters?** *Choose an item.*

5.1.6.5.1. **If yes, will you need to re-contact subjects? How will this be done?**

5.1.7. **Will this study create a data repository for future studies?** *NO*

5.1.7.1. **If yes, describe and/or provide the following:**

5.1.7.1.1. **The type of data (identified or de-identified) including what protected health elements are to be collected.**

5.1.7.1.2. **The source from which data will be collected (e.g., subjects, non-research data repositories, research data repositories, publicly available, VA source, non-VA source.**

5.1.7.1.3. **How and where the data will be stored (e.g., electronic, paper records, approved VA-owned or VA-leased space).**

5.1.7.1.4. **How the data will be transmitted, if applicable.**

5.1.7.1.5. **How the data will be secured during storage, use, and transmission both during the conduct of the research protocol and after the protocol is completed.**

5.1.7.1.6. **Plans to store data for future research. If the data is stored for future research, there must be a description of a research data repository, its location, and its security measures.**

5.1.7.1.7. **Plans to share with others including other researchers (VA and non-VA). If the data were collected through a research project, discussion of whether or not the original informed consent allowed for such reuse of the data and if the reuse is consistent with the HIPAA authorization that was obtained.**

5.1.7.1.8. **Justification for the use of any identifiers.**

5.1.7.1.9. **Justification that the data requested represent the minimum necessary to conduct the research.**

5.1.7.1.10. **A discussion of plans for obtaining informed consent and HIPAA Authorization, or for requesting the IRB to waive these requirements. If the investigator requests that the requirement for a HIPAA Authorization be waived, justification for this request must be included in information submitted to the IRB.**

5.1.7.1.11. **In addition to the above, provide a Standard Operating Procedures Manual for the data repository.** *Contact IRB office for additional details.*

5.2. **Subject Recruitment Methods**

5.2.1. **State how many subjects will be needed**: 180 total subjects, with 60 in each arm of the study.

5.2.2. **Who will be responsible for recruiting potential subjects? Provide titles of individuals.**

The study team will include:

Mitesh Patel, MD, MBA, MS – Principal Investigator
Anish Agarwal MD, MPH – Co-Investigator
Chalanda Evans – Project Manager
Victoria Hilbert – Project Manager
Kelsey Karpink – Clinical Research Coordinator
Rachel Djaraher – Clinical Research Coordinator

5.2.3. **How will initial contact with potential subjects be made? (*e.g., local clinics, physician referrals, letters to prospective subjects)***

Veterans receiving care at the CMCVAMC will be mailed a letter describing the study, and study contact information. They will have the ability to opt out from future communications if they like. If an individual is interested in the study, they will be given the opportunity to ask questions and instructed to sign up for the study on the Way to Health Platform. Then the veteran will be called to conduct oral consent and eligibility and baseline surveys. The study team will mail the veteran a copy of the consent form, a wearable device to track step counts and provide instructions to create an account by which they will transmit de-identified step data to the research team.

We have designed the study to be conducted remotely for recruitment, enrollment and the study design. This will allow Veterans to review the study and design in the comfort of their own homes with a member of the study team able to answer questions by phone. It will also reduce time burden any costs that would otherwise be placed on Veterans if he or she had to travel to the CMCVAMC for an in-person visit.

5.2.4. **Will you be using any of the following methods to recruit subjects? (Check all that apply.)**
N/A

X -Vinci ☐ **Local database for which subjects have NOT given prior permission to be contacted for Research.**

☐ **Personal contact with patients over whom you have direct/indirect oversight**

☐ **Provider (Clinician) Referrals of potential subjects**

5.2.5. **Indicate the types of recruitment/advertisement materials that will be used: Check all that apply. Submit copies of recruitment materials, for IRB review**.
X **Not applicable; none to be used**

☐**Fliers** ☐**Newspapers** ☐**Letters** ☐**Websites** ☐**Television**

☐**Radio** ☐**Audio** ☐**Video** ☐**Surveys**

☐**Other (Specify, e.g. employee newsletters)** ☐

5.2.6. **Non-Veteran Subjects will be given a copy of the Notice of Privacy Practices.** *NOT APPLICABLE*

5.3. **Compensation for Participation** - *YES* **If yes, complete the following**.

5.3.1. **Summarize any financial compensation that will be offered to subjects**.

All participants will receive $25 to enroll in the study (defined as being randomized and starting the intervention) and another $50 for completing the entire 20-week study. Participants randomized to the supportive social incentive intervention and financial incentive arm will have the opportunity to obtain up to an additional $120.

5.3.2. **Provide the schedule for compensation**.

5.3.2.1. **Per study visit or session.**
N/A

5.3.2.2. **Total amount for entire participation.**
$75 if subject completes the entire 20-week study or $195 for participants in the supportive social incentive intervention and financial incentive arm. .

5.3.3. **State how compensation will be provided:** *Voucher*

5.4. **Informed Consent Procedures**

5.4.1. **Indicate if informed consent will be obtained and/or if you are requesting a waiver of informed consent or waiver of documentation of informed consent.** *Consent to be obtained*

5.4.2. **If the research involves multiple phases, specify for which phases of the research the waiver(s) is/are being requested.**
N/A

5.4.3. **Describe circumstances, if any, that may need to be addressed in seeking informed consent (e.g., subjects with impaired decision making ability and the use of a legally authorized representative, etc.)**

Not applicable.

5.4.4. **If applicable, indicate how study personnel will be trained regarding human subjects protections requirements and how to obtain and document informed consent.**
All study personnel will have completed the required human subjects, HIPAA, and information security and privacy trainings at the VA.

5.4.5. **Inclusion/Exclusion Criteria: Describe the criteria that determine who will be included in or excluded from the study.**

5.4.5.1. **Inclusion Criteria**
1) Age 18 years or older; 2) interest in participating in a 20-week study using wearable devices to track step counts and increase physical activity; 3) body mass index of 25 or greater; 4) Smartphone or tablet compatible with application for the wearable activity tracking device.

5.4.5.2. **Exclusion Criteria**
1) Conditions that would make participation infeasible such as inability to provide informed consent, illiteracy or inability to speak, read, and write English; 2) conditions that would make participation unsafe such as pregnancy or being told by a physician not to exercise; 3) already enrolled in another study targeting physical activity; 4) any other medical conditions or reasons he or she is unable to participate in a physical activity study for 20 weeks.

5.5. <u>Withdrawal of Subjects</u>

5.5.1. **Describe how a subject can withdraw from the study**.
Subjects may withdraw at any point by informing the study team either by written mail or by phone.

5.5.2. **Describe any anticipated circumstances under which subjects will be withdrawn from the research without their consent**.
None.

5.5.3. **Describe the consequences of a subject's decision to withdraw from the research and the procedures for orderly termination of participation by the subject (e.g., the subject contacting the investigator for an end-of-study visit)**.
Patients choosing to withdraw early in the study will not receive the total compensation as described above. Patients are free to, at any time, contact the study team to be removed from the study.

5.6. <u>Potential Risk/Benefit Analysis</u>

5.6.1. **Potential Study Risks**

5.6.1.1. **Describe and assess all of the following risks that may be associated with the research**:

5.6.1.2. **Physical**
To minimize the chance for serious and unexpected adverse events, study participants will be screened through exclusion criteria for any health conditions that may be exacerbated by participating in a physical activity study. The program will use a gradual increase in physical activity during the first month that should pose little health risk to participants. Participants are given guidance on when to seek medical attention and a reporting protocol is in place to capture any changes in symptoms with physical activity.

5.6.1.3. **Psychological:** Not applicable.

5.6.1.4. **Social/Economic**
Each participant in the two intervention arms will identify a support partner (friend or family member) who will receive weekly updates by email on their progress in the

game. That support partner will be encouraged to help the participant to achieve their goals.

5.6.1.5. **Legal:** Not applicable.

5.6.1.6. **Loss of Confidentiality**

A potential risk of this study is a breach of participant confidentiality. We will minimize this risk by using secure data methods as described previously. Names and addresses will be stored in encrypted databases. These data will be viewable only by the respective participants and the study coordinator. All other members of the research team will be able to view only participant ID numbers. There will be no functionality in the web application to export a dataset with identifiable information. Even the study arms will be identified by code letters until both the statistician and PI agree that analysis is complete.

5.6.1.7. **Other, e.g. radiation, placebo, washout of medications:** Not applicable

5.6.1.8. **Assess the likelihood and seriousness of such risks**.: Not applicable

5.6.2. **Include a description of how anticipated risk will be minimized and include an analysis of risk vs. potential benefit.**

Anticipated risks of this study should be minimal and the risk/benefit ratio is very favorable. To minimize the chance for serious and unexpected adverse events, study participants will be screened through exclusion criteria for any health conditions that may be exacerbated by participating in this study. We have previously outlined the procedures that will be used to prevent a breach of participant data.

5.6.3. **Potential Study Benefits**

5.6.3.1. **Indicate potential benefits to be gained by the individual subjects, as well as benefit(s) that may accrue to society in general as a result of the planned work. If the subject will not receive any direct benefit, this fact must be stated here and in the consent form.**

Through participation in this study, each participant will have the potential to increase physical activity which could improve their health and reduce their risk for future disease. If this approach is effective, it could have tremendous benefits for society if adopted on a wide scale to help individuals. It is expected that other people will gain knowledge from this study and that participation could help understand how to effectively motivate individuals to change behavior. Participants may also receive no benefit from their participation in the study.

5.6.4. **Alternative Treatments Outside the Study**

5.6.4.1. **Describe alternatives available to the subject outside the research context. If there are no such alternatives, state that the alternative is not to participate in the research study**.

The alternative is not to participate in the research study.

5.7. **<u>Data Monitoring</u>**

5.7.1. **Will a Data and Safety Monitoring Board (DSMB) or Data Monitoring Committee (DMC) oversee the project?** *NO*

5.7.1.1. **If yes, provide contact information for the DSMB or DMC representative**.

5.7.1.2. **If no, describe the data and safety monitoring plan to be followed.**

The Principal Investigator will be responsible for monitoring the study. All participants will be given anticipatory guidance on when to seek medical attention. In addition, participants will be asked to report to the study team any injuries or medical care that they feel resulted from participation in the study. They can either call the study team or send an email. The research coordinator will call the participant to collect information regarding the issue and then the PI will review and determine whether it is ok to proceed, further investigation is needed, or the

participant should stop the study. For this study, there will be no stopping rules or endpoints and thus no planned interim analyses.

5.8. **<u>Reporting of Protocol Deviations, Adverse Events (AEs), Serious Adverse Events (SAEs), Breaches of Confidentiality, Unanticipated Adverse Device Effects (UADEs), and Unanticipated/Unexpected Problems</u>**

5.8.1. **Include procedures for reporting these events to the CMCVAMC IRB and sponsor**. Standard protocol will be followed for any events including reporting to the CMCVAMC IRB within 5 business days of discovery. We will use the CMCVAMC serious-adverse event form for reporting SAEs, UADEs, and any other unanticipated/unexpected problems. We will also use the CMCVAMC Protocol Deviation form for reporting any protocol deviations. Any true adverse events will be reported immediately.

5.9. **<u>Privacy and Confidentiality</u>**

5.9.1. **Describe whether the study will use or disclose subjects' Protected Health Information (PHI).**
In order to provide subjects with compensation for enrollment, the study will collect PHI. No PHI will be disclosed to any person outside of the research team.

5.9.2. **Check the PHI to be collected on all subjects for this research protocol.**

- X **Name**
- X **All geographic subdivisions smaller than a State, including street address, city, county, precinct, ZIP code, and their equivalent geographical codes, except for the initial three digits of a ZIP code if, according to the current publicly available data from the Bureau of the Census:**
  - **a. The geographic unit formed by combining all ZIP Codes with the same three initial digits contains more than 20,000 people; and**
  - **b. The initial three digits of a ZIP Code for all such geographic units containing 20,000 or fewer people are changed to 000.**
- X **All elements of dates (except year) for dates directly related to an individual, including birth date, admission date, discharge date, date of death; and all ages over 89 and all elements of dates (including year) indicative of such age, except that such ages and elements may be aggregated into a single category of age 90 or older.**
- X **Telephone numbers**
- ☐ **Fax numbers**
- X **Electronic mail addresses**
- X **Social Security/Medical Record Number**
- ☐ **Health plan beneficiary numbers**
- ☐ **Account Numbers**
- ☐ **Certificate/license numbers**
- ☐ **Vehicle identifiers and serial numbers, including license plate numbers**
- ☐ **Device identifiers and serial numbers**
- ☐ **Web universal resource locators (URLS)**
- ☐ **Internet protocol (IP) address numbers**
- ☐ **Biometric identifiers, including fingerprints and voiceprints**
- ☐ **Full-face photographic images and any comparable images**
- ☐ **Any other unique identifying number, characteristic, or code, unless otherwise permitted by the Privacy Rule for re-identification.**

☐ **HIV (testing or infectious disease) records** ☐ **Sickle cell anemia**

☐ **Drug Abuse Information** ☐ **Alcoholism or Alcohol Use**

5.10. **Information Security**

5.10.1. **List the data/information that will be stored (including signed, original informed consent and HIPAA authorization forms, if applicable, case report forms, etc.)**

Stored data and information will include: patient information including name, last 4 of SSN, date of birth, BMI, medical, problem list, signed, original informed consent forms, baseline questionnaires capturing participants' sociodemographic information, technology assessment, and current level of physical activity, any responses from follow-up phone interviews.

5.10.2. **Describe the steps that will be taken to secure the data (e.g., training, authorization of access, password protection, encryption, physical controls, Certificates of Confidentiality and separation of identifiers and data)**.

All study personnel will complete the required human subjects, HIPAA, and information security and privacy trainings at the VA. Each Veteran who enrolls in this study will be assigned a unique, random patient ID number generated for the purposes of this study. To protect each participant's identity, the link between the Veteran's name, last 4 of SSN, date of birth, and patient ID number will be a password protected file stored on a secure VA server and accessible only to research staff. In all subsequently created analytical files, participants will only be identified by their patient ID number, without inclusion of his/her name, SSN, or date of birth. Subject questionnaires will be either directly inputted into a computer database or written onto paper forms and then transferred to a database at a later time. Interview transcripts will be produced electronically and will be housed on a secure VA server as well. No results will be reported in a personally identifiable manner.

5.10.3. **Indicate how and where data/information will be stored, and specify pertinent security systems**.

The file linking Veterans' personal identifying information, patient database, questionnaire data, and interview transcripts will be password protected and stored on a secure VA server located within the VA firewall. Participant questionnaire data not containing PHI/PII will be stored in REDCap within the VA firewall.The server is physically located within the FITS computer room of the Philadelphia VAMC and networked within the VA Intranet. Thus, the servers have the same degree of physical and electronic protection afforded other VA computer systems, including antiviral protection and routine back-ups. FITS is responsible for managing the server hardware and software, including its physical and network security and connectivity, backup processes, operating system patches, and application management. Study data will be accessed using password protected computers that are not connected to the Internet and are entirely compliant with Federal Information Security Management Act (FISMA) standards. Paper records will be kept in a locked file cabinet in an electronically secured building. The likelihood of loss of confidentiality is very low given the information security and privacy requirements that are in place.

The study will use the "Way to Health" platform to provide close monitoring, feedback and reinforcement at a low cost to permit cost-effective flexible, scalable infrastructure. This platform has been used for a clinical trial at the CMCVAMC in the past. The platform was built at the University of Pennsylvania and aims to improve health behaviors and consists of a portal with links to variety of peripheral devices (e.g., scales, wearable devices, glucometers) for assessing health behaviors and outcomes; the capacity to communicate back to patients using interactive voice recording; and the ability to automate the delivery of feedback reports. For this study, step goals and reports will be sent to subjects (and if in "social incentive arm", reports will also be mailed to the support partner).

Once patients have consented to be in the study and have their data managed by *Way to Health (*WTH), the WTH platform adherence tracking information will be stored according to a unique, random, patient identifier generated for the purposes of the study. To assure that subject, physician and other informant confidentiality is preserved, individual identifiers (such as name and medical record number) are stored in a single password protected system that is accessible only to study research, analysis and IT staff. This system is hosted onsite at the University of Pennsylvania (UPenn) and is protected by a secure identification number (ID). Any datasets and computer files that leave the firewall will be stripped of all identifiers and individuals will be referred to by their study ID. The study ID will also be used on all analytical files.

The University of Pennsylvania Biomedical Informatics Consortium (BMIC) is the hub for the hardware and database infrastructure. The data collected for *Way to Health* based studies is stored in MySQL databases on a BMIC-operated blade server environment devoted specifically to *Way to Health*. The data center is housed in the Information Systems and Computing at 3401 Walnut Street. All data are stored in a single relational database, allowing researchers to correct mistakes. Every SQL transaction, including accessing and changing data is logged for auditing purposes. Data are entered into the database through several different mechanisms. A program specialist will enter subjects' personal information and responses to survey questions through a PHP-based web interface. Data from monitoring devices are uploaded automatically. Datasets are blinded of all personally identifiable information when exported for analysis. The web application automatically removes all identifiers when a member of the research team requests an analytic dataset. The only people with access to identifiable participant information are pre-specified Research Coordinators responsible for contacting participants. Personal information and research data will be stored in separate SQL tables and will be linked by a computer-generated ID number. All data for this project will be stored on the secure/firewalled servers for the BMIC Data Center, in data files that will be protected by multiple password layers. These data servers are maintained in a guarded facility behind several locked doors, with very limited physical access rights. They are also cyber-protected by extensive firewalls and multiple layers of communication encryption. Electronic access rights are carefully controlled by UPenn system managers. We believe this multi-layer system of data security, identical to the system protecting the University of Pennsylvania Health System medical records, greatly minimizes the risk of loss of privacy.

5.10.4. **Will PHI be transmitted or transported outside of CMCVAMC?** *NOT APPLICABLE*
If yes, complete sections 5.10.4.1 through 5.10.4.3, and an Off-site Storage/Transfer of Research Data form. <u>If no, go directly to section 5.11.</u>

5.10.4.1. **Does the informed consent document and Authorization for Use & Release of Individually Identifiable Health Information for Veterans Health Administration (VHA) Research form disclose entities/individuals to which/whom PHI will be transported or transmitted?** *Choose an item.*

5.10.4.2. **Specify entities/individuals outside CMCVAMC to which/whom data will be disclosed, the justification for such disclosure and the authority, and how they will access it**.

5.10.4.3. **List the data/information that will be transmitted or transported, and specify how data will be transported or transmitted from one location to another and how it will be protected during transmission or transportation outside of CMCVAMC.**

5.11. Data Management Access Plan

5.11.1. DMAP form **must** be included with all **initial** submissions. The DMAP form can be found on the Research and Development SharePoint site.

5.12. <u>Communication Plan</u>

5.12.1. **Include plan for ensuring that the study is conducted according to the IRB-approved protocol.**
All study personnel will meet regularly to ensure that the study is conducted according to the IRB approved protocol. At these meetings, they will discuss unforeseen challenges as they arise and together create a plan for troubleshooting these issues within the confines of the IRB approved protocol.

5.13. **Is this Study Investigating the Use of a Drug or Biological Agent?** *NO* **If yes, complete the rest of this section. If no, go directly to section 6, unless 5.13 applies.**

5.13.1. **Specify if the drug or biological agent is:**

5.13.1.1. **FDA approved:** *Choose an item.*

5.13.1.2. **Used for off-label purposes:** *Choose an item.*

5.13.2. **Include the FDA Investigational New Drug (IND) number for all non-FDA approved and off-label drugs, biological agents or nutritional supplements. If not applicable state, "Not Applicable."**

5.13.3. **Provide all relevant information about the drug, including pre-clinical data.**

5.13.4. **Explain any wash-out periods, rescue medications permitted and any type of medications not permitted while enrolled in the study.**

5.13.5. **Describe blinding and un-blinding procedures.**

5.13.6. **Include the dosage, route of administration, previous use, and the safety and efficacy information on any drug used for research purposes.**

5.13.7. **Describe rationale for the dosage in this study.**

5.13.8. **Justify why the risks are reasonable in relation to anticipated benefits and/or knowledge.**

5.13.9. **Describe where drug preparation will be done.**

5.13.10. **All drugs for CMCVAMC subjects must be dispensed through the VA investigational pharmacy.**

5.13.11. **Describe where the study treatment will be administered.**

5.13.12. **Describe plan for tracking a non-compliant treatment study subject.**

5.13.13. **Describe the process for the storage, security, dispensing and return of an investigational drug.**

5.13.14. **Has this protocol has been submitted to the Medical Center's Pharmacy and Therapeutics Committee?** *Choose an item.*

5.14. **Is this Study Investigating the Use of a Device** - *NOT APPLICABLE* **If yes, complete the rest of this section. If no, go directly to section 6.**

5.14.1. **The Investigational Device Exemption (IDE) number must be submitted for all significant risk devices and if an IDE exists for a non-significant risk device.**

5.14.2. **Significant Risk or Non-significant Risk - If a device is not approved by the FDA, specify whether or not the sponsor has determined this device to be a "significant risk" or "non-significant risk" as defined by the FDA.**

5.14.3. **Provide all relevant information about the device.**

5.14.4. **Describe blinding and un-blinding procedures.**

5.14.5. **Specify if device is:**

5.14.5.1. **FDA approved:** *Choose an item.*

5.14.5.2. **Used for off-label purposes:** *Choose an item.*

5.14.6. **Explain if the investigational device will be delivered and/or stored by the Principal Investigator or Pharmacy Service.**

5.14.7. **Describe the process for the storage, security, dispensing and return of an investigational device.**

5.14.8. **For research involving an investigational device, describe the SOP or plan for device control.**

5.14.9. **Address how the device will be stored in such a way that only research staff associated with the protocol will have access to the device.**

5.14.10. **Describe measures that will be put into place to ensure that the device will only be used in subjects of this research protocol.**

Section 6: Resources and Personnel

6.1. **Include where and by whom the research will be conducted.**

The study will be coordinated out of the CMCVAMC and subjects will wear tracking devices during their normal activity throughout the study time period. The team includes: Mitesh Patel, MD, MBA, MS – Principal Investigator , Anish Agarwal MD, MPH – Co-Investigator, Chalanda Evans – Project Manager,

Victoria Hilbert – Project Manager, Kelsey Karpink – Clinical Research Coordinator, and Rachel Djaraher – Clinical Research Coordinator.

6.2. **Provide a brief description of each individual's role in the study. Indicate who will have access to protected health information and who will be involved in recruiting subjects; obtaining informed consent; administering survey/interview procedures; and performing data analysis**.
The team includes: Mitesh Patel, MD, MBA, MS – Principal Investigator , Anish Agarwal MD, MPH – Co-Investigator, Chalanda Evans – Project Manager, Victoria Hilbert – Project Manager, Kelsey Karpink – Clinical Research Coordinator, and Rachel Djaraher – Clinical Research Coordinator. These team members will only have access to PHI and will be working collectively to recruit subjects, obtain consents and administer surveys. Mitesh Patel and Anish Agarwal will perform data analysis.

6.3. **If applicable, provide information on any services that will be performed by contractors, including what is being contracted out and with whom**.
Not applicable.

6.4. **If applicable, provide information on any Memoranda of Understanding (MOUs) or Data Use Agreements (DUAs) that are being entered into, including with whom and for what reason**.
Not applicable.

Section 7: Genetic Testing

7.1. **Does the project involve genetic testing?** *Not Applicable, SKIP TO SECTION 8*

7.2. **Will specimens be kept for future, unspecified use?** *Choose an item.*

7.3. **Will samples be made anonymous to maintain confidentiality?** *Choose an item.* (*If there is a link, it is not anonymous. Coding is not anonymous.)*

7.4. **Will specimens be destroyed after the project-specific use is completed?** *Choose an item.*

7.5. **Will specimens be sold in the future?** *Choose an item.*

7.6. **Will subjects be paid for their specimens now or in the future?** *Choose an item.*

7.7. **Will subjects be informed of the results of the specimen testing?** *Choose an item.*

7.8. **Are there any implications for family members based on specimen testing results?** *Choose an item.*
7.8.1. I**f answer to section 7.8 is yes, they may be subjects**.

7.9. **Will subjects be informed of results obtained from their DNA?** *Choose an item.*

7.10. **Explain if the study is looking for an association between a genetic marker and a specific disease or condition, but at this point it is not clear if the genetic marker has predictive value**.

7.11. **Describe if the study is based on the premise that a link between a genetic marker and a specific disease or condition is such that the marker is clinically useful in predicting the development of that specific disease or condition.**

7.12. **Will the subject be notified of the results and the provision for genetic counseling?** *Choose an item.*

Section 8: International Research

8.1. **Does this study involve international research?** *NOT APPLICABLE* **If no, go directly to section 9.**

Section 9: Statistical Analysis

9.1. **Include statistical power calculations and the assumptions made in making these calculations.**

This study has been powered for two phases of hypothesis testing. In the first phase, we will compare each of the two intervention arms to control. We estimate that a sample of 180 participants allocated in a 1:1:1 distribution, will ensure at least 80% power to detect a 900-step difference between each intervention arm and control, with a standard deviation of 1500 steps. This calculation assumes a 10% missing data rate and a conservative Bonferroni adjustment of the type I error rate with a 2-sided alpha of 0.025. In the second phase, we will compare successful intervention arms to each other. We expect that the magnitude of difference between intervention arms will be less than that of successful intervention arms compared to control. For this second phase of analyses will use a conservative Bonferroni adjustment of the type I error rate with a 2-sided alpha of 0.017 to adjust for up to 3 comparisons. In 2012, more than 57,500 veterans enrolled for care at CMCVAMC and comprised nearly 463,000 visits. Since 70% of veterans are overweight or obese, nearly 40,000 veterans may be eligible for this study. Based on this data and prior studies, we estimate that we can fill the study within 6 months. We will plan to oversample women and minorities in the recruitment process.

9.2. **Define plans for data and statistical analysis, including key elements of the statistical plan, stopping rules and endpoints.**

Data from the clinical trial will be analyzed using statistical software in SAS or R. In our primary analyses, we will multiple imputation for missing data and step values less than 1000. We will use linear mixed effects models to compare the change in mean daily step count from baseline to intervention period (weeks 5 to 12), adjusting for baseline step count and time. To test of the robustness of our findings we will also evaluate models using collected data without imputation. We will conduct similar models for the secondary outcome of change in mean daily steps from baseline to follow-up and use logistic models for the secondary outcomes of proportion of participant-days meeting goal during intervention and follow-up. All hypothesis tests will use a conservative Bonforroni adjustment as described in the power calculation.

9.3. **Provide sample size determination and analysis (include anticipated rate of screen failures, study discontinuations, lost to follow-up, etc.)**

See above.

9.4. **Describe how, where and by whom the data will be analyzed**.

The data will be analyzed by Mitesh Patel and Anish Agarwal with the help of CHERP faculty at the VA.

Section 10: References

1. Ischemic Heart Disease Quality Enhancement Research Initiative. U.S. Department of Veterans Affairs. June 2014. Available online at: http://www.queri.research.va.gov/about/impact_updates/IHD.pdf. Accessed January 26, 2017

2. Lee IM, Shiroma EJ, Lobelo F, Puska P, Blair SN, Katzmarzyk PT, Lancet Physical Activity Series Working Group. Effect of physical inactivity on major non-communicable diseases worldwide: an analysis of burden of disease and life expectancy. Lancet. 2012;380(9838):219-29.

3. Littman AJ, Forsberg CW, Koepsell TD. Physical activity in a national sample of veterans. Med Sci Sports Exerc. 2009;41(5):1006-13.

4. Patel MS, Asch DA, Volpp KG. Wearable Devices as Facilitators, Not Drivers, of Health Behavior Change. JAMA. 2015;313(5):459-60.

5. Evidence-based Synthesis Program. The impact of wearable motion sensing technologies on physical activity: A systematic review. Department of Veterans Affairs Health Services Research & Development Service. September, 2015.

6. Loewenstein G, Brennan T, Volpp KG. Asymmetric paternalism to improve health behaviors. JAMA. 2007;298(20):2415-7.

7. Patel MS, Asch DA, Rosin R, Small DS, Bellamy SL, Heuer J, Sproat S, Hyson C, Haff N, Lee SM, Wesby L, Hoffer K, Shuttleworth D, Taylor D, Hilbert V, Zhu J, Yang L, Wang X, Volpp KG. Framing financial incentives to increase physical activity among overweight and obese adults: a randomized, controlled trial. Ann Intern Med. 2016;164(6):385-394.

8. Patel MS, Asch DA, Rosin R, Small DS, Bellamy SL, Eberbach K, Walters KJ, Haff N, Lee SM, Wesby L, Hoffer K, Shuttleworth D, Taylor D, Hilbert V, Zhu J, Yang L, Wang X, Volpp KG. Individual versus team-based financial incentives to increase physical activity: a randomized, controlled trial. JGIM. 2016;31(7):746-754.

9. Patel MS, Volpp KG, Rosin R, Bellamy SL, Small DS, Fletcher MA, Osman-Koss R, Brady JL, Haff N, Lee SM, Wesby L, Hoffer K, Shuttleworth D, Taylor DH, Hilbert V, Zhu J, Yang L, Wang X, Asch DA. A randomized trial of social comparison feedback and financial incentives to increase physical activity. Am J Health Promot. 2016;30(6):416-424.

10. Richlie D, Winters S, Prochazka AV. Dyslipidemia in veterans: Multiple risk factors may break the bank. Arch Intern Med. 1991;151:1433–1436.

11. Asch DA, Rosin R. Engineering social incentives for health. N Engl J Med. 2016; 375:2511-2513.

12. Christakis NA, Fowler JH. The spread of obesity in a large social network over 32 years. N Engl J Med. 2007;357(4):370-9.

13. Christakis NA, Fowler JH. The collective dynamics of smoking in a large social network. N Engl J Med. 2008;358(21):2249-58.

14. Jackson SE, Steptoe A, Wardle J. The Influence of Partner's Behavior on Health Behavior Change: The English Longitudinal Study of Ageing. JAMA Intern Med. 2015;175(3):385-92.

15. Case MA, Burwick HA, Volpp KG, Patel MS. Accuracy of Smartphone Applications and Wearable

Devices for Tracking Physical Activity Data. JAMA. 2015;313(6):625-626.

16. Das SR, Kinsinger LS, Yancy WS, Wang A, Ciesco E, Burdick M, Yevich SJ. Obesity prevalence among veterans at Veterans Affairs medical facilities. Am J Prev Med. 2005;28(3):291-4.

17. Asch DA, Volpp KG. On the Way to Health. LDI Issue Brief. 2012;17(9).

18. Ariely D, Wertenbroch K. Procrastination, deadlines, and performance: self-control by precommitment. Psychol Sci. 2002;13(3):219–24.

19. Rogers T, Milkman KL, Volpp KG. Commitment Devices: Using Initiatives to Change Behavior. JAMA. 2014;311(20):2065-6.
