# Supplementary material for: Effect of Gamification With and Without Financial Incentives to Increase Physical Activity Among Veterans Classified as Having Obesity or Overweight: A Randomized Clinical Trial
Source: JAMA Netw Open. 2021 Jul 9;4(7):e2116256. doi: 10.1001/jamanetworkopen.2021.16256 (PMC8271358; doi:10.1001/jamanetworkopen.2021.16256)
Supplement: Supplement 2. — eTable 1. Missing Data Rates by Arm and Study Period eTable 2. Adjusted Differences in Outcomes Without Multiple Imputation eTable 3. Patient Perceptions of Trial Experience From End-of-Study Surveys [file jamanetwopen-e2116256-s002.pdf]

Supplemental Online Content

Agarwal AK, Waddell KJ, Small DS, et al. Effect of gamification with and without financial incentives to increase physical activity among veterans classified as having obesity or overweight: a randomized clinical trial. *JAMA Netw Open*. 2021;4(7):e2116256. doi:10.1001/jamanetworkopen.2021.16256

eTable 1. Missing Data Rates by Arm and Study Period

eTable 2. Adjusted Differences in Outcomes Without Multiple Imputation

eTable 3. Patient Perceptions of Trial Experience From End-of-Study Surveys

This supplemental material has been provided by the authors to give readers additional information about their work.

© 2021 Agarwal AK et al. *JAMA Network Open.*

eTable 1: Missing Data Rates by Arm and Study Period

Variable	Control	Gamification with Social Support	Gamification with Social Support and Loss-Framed Financial Incentives
**Physical Activity (steps per day)**			
**Weeks 1-12**			
Missing or 0	523/5040 (10.4%)	800/5040 (15.8%)	715/5040 (14.1%)
Value 1 to 999	176/5040 (3.5%)	175/5040 (3.5%)	147/5040 (2.9%)
Event missing	39/5040 (0.7%)	2/5040 (0.03%)	39/5040 (0.7%)
**Weeks 1-4**			
Missing or 0	90/1680 (5.5%)	145/1680 (8.6%)	122/1680 (7.3%)
Value 1 to 999	49/1680 (2.9%)	46/1680 (2.7%)	36/1680 (2.1%)
Event missing	24/1680 (1.4%)	2/1680 (0.1%)	8/1680 (0.5%)
**Weeks 5-12**			
Missing or 0	433/3360 (12.9%)	655/3360 (19.5%)	593/3360 (17.6%)
Value 1 to 999	127/3360 (3.8%)	129/3360 (3.8%)	111/3360 (3.3%)
Event missing	15/3360 (0.5%)	0/3360 (0.0%)	31/3360 (0.9%)
**Weeks 13-20**			
Missing or 0	1021/3360 (30.3%)	1152/3360 (34.3%)	1276/3360 (38.0%)
Value 1 to 999	116/3360 (3.5%)	122 (3.6%)	110/3360 (3.3%)
Event missing	0/3360 (0.0%)	2/3360 (0.1%)	51/3360 (1.5%)

*Event missing periods defined as events where participants lost their device/charger and were waiting for a replacement, was hospitalized, injured, or other reasons that may have impacted their daily step count or resulted in missing activity data.

© 2021 Agarwal AK et al. *JAMA Network Open.*

eTable 2: Adjusted Differences in Outcomes Without Multiple Imputation

Variable	Timeline	Control	Gamification with Social Support	Gamification with Social Support and Loss-Framed Financial Incentives
**Steps per day**	**Baseline**			
	Mean (SD)	5881 (2038)	6012 (2494)	6105 (2320)
	**Intervention Period (Weeks 5 to 12)**			
	Difference relative to Control (95% CI)	-	463 (-324, 1250)	1123 (317, 1928)
	P Value	-	*0.48*	*0.02*
**Steps per day**	**Baseline**			
	Mean (SD)	6195 (3767)	6012 (2494)	6105 (2320)
	**Follow-up Period (Weeks 13 to 20)**			
	Difference relative to Control (95% CI)	-	-211 (-1142, 720)	80 (-852, 1012)
	P Value	-	*0.89*	*0.98*
**Proportion of Days Achieving Goal**	**Intervention Period (Weeks 5 to 12)**	0.25		
	Difference relative to Control (95% CI)	-	0.22 (0.18, 0.25)	0.34 (0.30, 0.39)
	P Value	-	*<0.001*	*<0.001*
**Proportion of Days Achieving Goal**	**Follow-up Period (Weeks 13 to 20)**	0.26		
	Difference relative to Control (95% CI)	-	0.07 (0.04, 0.10)	0.13 (0.09, 0.17)
	P Value	-	*<0.001*	*<0.001*

© 2021 Agarwal AK et al. *JAMA Network Open.*

eTable 3: Patient Perceptions of Trial Experience From End-of-Study Surveys

Survey Measures (n)	Control	Gamification with Social Support	Gamification with Social Support and Loss-Framed Financial Incentives)
**Follow-up Survey**			
Previously heard of device			
Yes	50 (83.3%)	48 (80.0%)	42 (72.4%)
No	5 (8.3%)	9 (15.0%)	10 (17.2%)
Missing	5 (8.3%	3 (5.0%)	8 (13.8%)
Like Device (Love it)	30 (50.0%)	25 (41.7%)	24 (41.4%)
Frequency of Device Wear			
Never	12 (20%)	10 (16.7%)	14 (24.1%)
1-3 hours per day	1 (1.7%)	2 (3.3%)	0 (0.0%)
3-6 hours per day	0 (0.0%)	2 (3.3%)	1 (1.7%)
6-10 hours per day	4 (6.7%)	0 (0.0%)	1 (1.7%)
Over 10 hours per day	3 (5.0%)	6 (10.0%)	4 (6.9%)
24 hours per day	35 (58.3%)	37 (61.7%)	32 (55.2%)
Missing	5 (8.3%)	3 (5.0%)	8 (13.8%)
Importance of battery life (Very important)	36 (60.0%)	45 (75.0%)	34 (58.6%)
Enjoyed using Fitbit to track step counts (Strongly agree)	45 (75.0%)	42 (70.0%)	42 (72.4%)
Enjoyed participating in the study (Strongly agree)	45 (75.0%)	42 (70.0%)	46 (79.3%)
Study helped to increase physical activity level	30 (50.0%)	38 (63.3%)	44 (75.9%)

© 2021 Agarwal AK et al. *JAMA Network Open.*
