# Supplementary material for: Effect of Gamification With and Without Financial Incentives to Increase Physical Activity Among Veterans Classified as Having Obesity or Overweight: A Randomized Clinical Trial
Source: JAMA Netw Open. 2021 Jul 9;4(7):e2116256. doi: 10.1001/jamanetworkopen.2021.16256 (PMC8271358; doi:10.1001/jamanetworkopen.2021.16256)
Supplement: Supplement 3. — Data Sharing Statement [file jamanetwopen-e2116256-s003.pdf]

Data Sharing Statement

Agarwal. Effect of Gamification With and Without Financial Incentives to Increase Physical Activity Among Veterans Classified as Having Obesity or Overweight: A Randomized Clinical Trial. *JAMA Netw Open*. Published July 09, 2021.
doi:10.1001/jamanetworkopen.2021.16256

Data
Data available: No
